# FABP4 activates the JAK2/STAT2 pathway via Rap1a in the homocysteine-induced macrophage inflammatory response in *ApoE*^*−/−*^ mice atherosclerosis

**DOI:** 10.1038/s41374-021-00679-2

**Published:** 2021-11-01

**Authors:** Lingbo Xu, Huiping Zhang, Yanhua Wang, Anning Yang, Xiaoyan Dong, Lingyu Gu, Dayue Liu, Ning Ding, Yideng Jiang

**Affiliations:** 1 Department of Pathophysiology, School of Basic Medical Sciences, Ningxia Medical University, Yinchuan, 750004 China; 2 National Health Commission Key Laboratory of Metabolic Cardiovascular Diseases Research, Ningxia Medical University, Yinchuan, 750004 China; 3 Ningxia Key Laboratory of Vascular Injury and Repair Research, Ningxia Medical University, Yinchuan, 750004 China; 4 Prenatal Diagnosis Center of Ningxia Medical University General Hospital, Yinchuan, 750004 China; 5 Department of Gynecology, General Hospital of Ningxia Medical University, Yinchuan, 750004 China

**Keywords:** Extracellular signalling molecules, Cell death and immune response

## Abstract

Atherosclerosis is a chronic inflammatory vascular disease, and inflammation plays a critical role in its formation and progression. Elevated serum homocysteine (Hcy) is an independent risk factor for atherosclerosis. Previous studies have shown that fatty acid binding protein 4 (FABP4) plays an important role in macrophage inflammation and lipid metabolism in atherosclerosis induced by Hcy. However, the underlying molecular mechanism of FABP4 in Hcy-induced macrophage inflammation remains unknown. In this study, we found that FABP4 activated the Janus kinase 2/signal transducer and activator of transcription 2 (JAK2/STAT2) pathway in macrophage inflammation induced by Hcy. Of note, we further observed that ras-related protein Rap-1a (Rap1a) induced the Tyr416 phosphorylation and membrane translocation of non-receptor tyrosine kinase (c-Src) to activate the JAK2/STAT2 pathway. In addition, the suppressor of cytokine signaling 1 (SOCS1)—a transcriptional target of signal transducer and activator of transcription (STATs) inhibited the JAK2/STAT2 pathway and Rap1a expression via a negative feedback loop. In summary, these results demonstrated that FABP4 promotes c-Src phosphorylation and membrane translocation via Rap1a to activate the JAK2/STAT2 pathway, contributing to Hcy-accelerated macrophage inflammation in *ApoE*^−/−^ mice.

## Abstract (web summary)

**A proposed regulatory model of FABP4 in Hcy-induced macrophage inflammation in atherosclerosis of** ***ApoE***^**−/−**^ **mice.** FABP4 activates the JAK2/STAT2 pathway via Rap1a in Hcy-induced macrophage inflammatory response and atherosclerosis in *ApoE*^*−/−*^ mice, which is attributed to Rap1a-dependent promotion of the c-Src phosphorylation at Tyr416 and membrane translocation. SOCS1 has a negative regulatory role in FABP4-dependent activation of the JAK2/STAT2 pathway and Rap1a in macrophage inflammatory response induced by Hcy.

## Graphical abstract

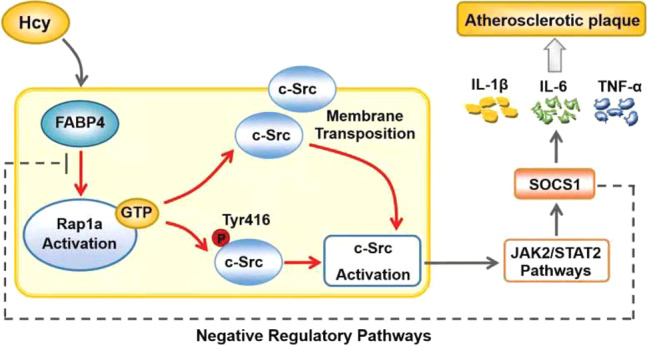

## Introduction

Among vascular diseases, atherosclerosis is one of the leading causes of death worldwide^1^. Numerous mechanisms have been proposed to explain the pathogenesis of homocysteine (Hcy)-associated atherosclerosis, including vascular inflammation and dyslipidemia^2^. However, the detailed mechanism of macrophage inflammation in atherosclerosis induced by Hcy remains to be elucidated.

Fatty acid-binding protein 4 (FABP4), a member of fatty acid-binding proteins (FABPs) family, is predominantly expressed in macrophages and adipocytes; it plays a critical role in lipid metabolism and inflammatory processes^3^. Previous studies have shown that circulating FABP4 may be a useful additional biomarker to evaluate patients with stable peripheral vascular diseases at risk of major cardiovascular complications^4^. Besides, it has been observed that the expression of inflammatory cytokines TNF-α, IL-1β, and IL-6 is significantly reduced in FABP4-deficient macrophages in atherosclerosis^5^. Our previous studies have shown that FABP4 hypomethylation accelerates cholesterol accumulation in atherosclerosis induced by Hcy, suggesting that FABP4 may serve as a marker of atherosclerosis^6^.

The Janus kinase/signal transducers and activators of transcription (JAK/STAT) pathway regulates macrophages development and function by participating in signal transduction of numerous cytokines, growth factors, and hormones^7^. The activation of JAK/STAT pathway can induce the expression of cytokine signal transduction (SOCS) protein, which in turn negatively regulates JAK/STAT pathway^8^. Therefore, the aim of the present work was to investigate the effect of FABP4 on regulating the JAK2/STAT2 pathway in macrophage inflammation in atherosclerosis.

Ras-related protein Rap-1a (Rap1a) is a member of small GTPases, and it is considered a key regulator of leukocyte and endothelial cell responses^9^. One common mechanism utilized by small GTPases to regulate cellular function is to cycle between the inactive GDP-bound state and the active GTP-bound state^10^. Besides, it links activation of non-receptor tyrosine kinases such as sarcoma (c-Src) gene with downstream effector pathways^11^. c-Src is a membrane-associated non-receptor type tyrosine kinase that regulates cell growth, adhesion, and migration^12^. c-Src activation includes phosphorylation and membrane translocation^13^. On the one hand, the main phosphorylation sites of c-Src include tyrosine 416, whose autophosphorylation results in activation, and tyrosine 527, whose phosphorylation by C-terminal Src kinase leads to inhibition of c-Src. On the other hand, c-Src binds to cell membranes, thus transferring signals from extracellular to intracellular space^14^.

In this study, we showed that FABP4 activated the JAK2/STAT2/SOCS1 pathway via Rap1a in macrophages treated with Hcy. In addition, Rap1a facilitated c-Src phosphorylation at Tyr416 and membrane translocation in macrophage inflammation. Our findings offer new insights into the mechanism of Hcy-induced macrophage inflammation, which may provide a novel FABP4-based therapeutic approach to atherosclerosis.

## Materials and methods

### Reagents

The antibodies anti-FABP4 (number ab92501), anti-JAK1 (number ab47435), anti-JAK3 (number ab45141), anti-STAT1 (number ab30645), anti-STAT3 (number ab76315), anti-SOCS1 (number ab9870), anti-SOCS2 (number ab3692), and anti-Rap1a (number ab197673) were purchased from Abcam (Cambridge, MA). Anti-Src (number 2108S), anti-p-Src family (Tyr416 number 6943S), anti-STAT2 (number 72604S), anti-JAK2 (number 3230S), anti-p-JAK2 (Y1007/1008, number 3771S), and anti-p-STAT2 (number 88410S) antibodies were obtained from Cell Signaling Technology (Danvers, MA), while anti-β-actin (number sc-8432) was obtained from Santa Cruz Biotechnology (Santa Cruz, CA).

### Animal model and serum Hcy levels

All experimental procedures and animal care followed the Detailed Rules and Regulations of Medical Animal Experiments Administration and Implementation, Ministry of Public Health, PR China. Six-week-old male apolipoprotein-E-knockout (*ApoE*^−/−^) mice with C57BL/6J background purchased from the Charles River Laboratories company (Beijing, China) and fed in specified pathogen-free conditions were randomly divided into two groups and treated as follows: (1) *ApoE*^−/−^ +NC: *ApoE*^−/−^ mice were fed regular diet; (2) *ApoE*^−/−^ +HMD: *ApoE*^*−/−*^ mice were fed 1.7% methionine diet^15,16^. Blood was collected from the orbital sinus of anesthetized mice; following centrifuge at 3000 × *g* for 10 min at 4 °C after incubation at room temperature for 30 min, Hcy concentrations in serum were measured by automatic biochemistry analyzer (SIEMENS, Germany).

### Assessment of atherosclerotic lesions

The *ApoE*^*−/−*^ mice and cotton ball of ether were put into the sealed anesthesia bottle, and warmed ultrasound transmission gel was placed on the mice chest. The mechanical transducer (Vevo 770, Visual Sonics, Toronto Canada) was set at 40 MHz, and the Real-Time Micro Visualization Scanhead (RMV 704) was used for intima–media thickness (IMT) and blood velocity measurements. Subsequently, the *ApoE*^*−/−*^ mice aortas were flushed with saline and embedded in optimum cutting temperature compound. Four-micrometer-thick frozen sections were stained with hematoxylin and eosin (HE) and Oil Red O staining. The percent lesion area of the aortic sinus was calculated by ImageJ software (Media Cybernetics). Six mice per group were measured by a single observer blinded to the experimental protocol and were used for analysis.

### Immunofluorescence staining

The aortic roots of *ApoE*^*−/−*^ mice and macrophages were fixed with cold acetone for 30 min, blocked with goat serum at 4 °C, and then incubated with primary antibodies at 4 °C overnight. Subsequently, the specimens were incubated with corresponding Cy3- and FITC-conjugated secondary antibody at 37 °C for 1 h, while nuclei were stained with 4,6-diamino-2-phenyl indole (DAPI) for 5 min at room temperature. Digital images were acquired with OLYMPUS FV3000 confocal laser scanning microscope (Tokyo, Japan).

### Cell culture

Human acute monocytic leukemia cell line (THP-1) was purchased from the American Type Culture Collection (ATCC; USA). The cells were cultured in RPMI 1640 (Gibco, USA) supplemented with 10% fetal bovine serum (Gibco, USA) and 100 U/ml penicillin–streptomycin (Gibco, USA) at 37 °C under a humidified 5% CO_2_ atmosphere. THP-1 cells were treated with 100 nmol/l phorbol 12-myristate 13-acetate (PMA) for 48 h to induce the macrophage model. After that, macrophages were divided into two groups: Control group, where we added 7% RPMI 1640 medium without Hcy for 48 h; and Hcy group, which was treated with 100 μmol/L Hcy for 48 h and medium was changed every 24 h. Hcy was dissolved in 7% RPMI 1640 medium until the final concentration of 100 μmol/L.

### Cell adenovirus transduction

Adenovirus vectors encoding FABP4 (Ad-FABP4) and Adenovirus vectors encoding GFP (Ad-GFP) were packaged and purified by Gene-Pharma (Shanghai, China). The adenoviruses were diluted using RPMI 1640 and added into the medium supernatant of macrophages at a multiplicity of infection of 100 and incubated for more than 48 h. Transduction with Ad-GFP alone served as a control for FABP4 overexpression. Fluorescent signals were observed under a fluorescence microscope to evaluate the efficiency of transduction.

### Gene microarray assay

Total RNA was extracted from macrophages by RNeasy mini kits (Qiagen, China) and reversed to double-stranded cDNA by MEG Ascript TM T7 Kit (Ambion, USA). The fragment of double-stranded cDNA was hybridized to U95Av2 gene chip (Affymetrix) and washed following the manufacturer’s instructions. Each hybrid Affymetrix gene chip was scanned at 570 nm using an argon-ion laser scanner (Affymetrix). Affymetrix custom image analysis software (Gene chip version 3.1) was used for initial absolute analysis and comparative analysis of data images. The detection specificity of the Gene chip was 1/100,000 copies. The gene data analysis was based on GenBank Database (http://www.ncbi.nim.nih.gov) or Gene Cards Database (http://bioinf.weizmann.ac.il/cards/index.html).

### Quantitative real-time PCR (qRT-PCR)

Total RNA was extracted and reverse-transcribed using Takara RT-PCR kit (Dalian, China). The primers were purchased from Sangon Biotech (Shanghai, China). qRT-PCR was performed using SYBR Green Real-Time PCR Master Mix Kit (Toyobo, Japan) on an FTC3000 RT-qPCR System (Funglyn Biotech Inc, Toronto, Canada). The expression level of the target gene was calculated using 2^−ΔΔCT^ method. Primer sequences are listed in Table 1.

**Table 1:** Primer sequences for real-time PCR analyses.

Gene	GenBank	Primer sequence, 5′ to 3′	Product size (bp)	Annealing temperature
GAPDH	NM_001289745	F: AACGGATTTGGTCGTATTGCA	215	60 °C
		R: CGCTCCTGGAAGATGGTGAT		
FABP4	NM_001442.3	F: CAGGAAAGTGGCTGGCATGGC	293	60.3 °C
		R:CTCTCGTGGAAGTGACGCCTTTC		
JAK2	NM_001322194	F: TTATTCAGCAATTCAGCCAATG	215	58.2 °C
		R: TCACTTTCTTTATGTTTCCCTCTT		
STAT2	NM_198332	F: ATGGGCTTTGTGAGTCGG	170	56.5 °C
		R: GCCTTCGTGTACGGTTGC		
SOCS1	NM_003745	F: CGACACGCACTTCCGCACATT	158	58.5 °C
		R: GGGTCCCGAGGCCATCTTCAC		
Rap1a	NM_001010935.3	F: CGTGAGTACAAGCTAGTGGTCC	166	61.4 °C
		R: CCAGGATTTCGAGCATACACTG		
CCL8	NM_005623	F:TGGAGAGCTACACAAGAATCACC	133	61.4 °C
		R:TGGTCCAGATGCTTCATGGAA		
CXCL10	NM_001565	F:GTGGCATTCAAGGAGTACCTC	198	60.8 °C
		R:TGATGGCCTTCGATTCTGGATT		
IL-1β	NM_000576.3	F: GCGGCATCCAGCTACGAATCTC	244	55.3 °C
		R: CGGAGCGTGCAGTTCAGTGATC		
IL-6	NM_000600.5	F: CTCACCTCTTCAGAACGAATTG	149	60.8 °C
		R: CCATCTTTGGAAGGTTCAGGTTG		
TNF-α	NM_000594.4	F: CCTCTCTCTAATCAGCCCTCTG R: GAGGACCTGGGAGTAGATGAG	220	60.5 °C

*F* forward primer, *R* reward primer.

### Western blot

Total protein was extracted from macrophages using protein lysis solution. Protein lysis solution was separated by SDS-PAGE and then electrotransferred onto polyvinylidene fluoride membranes (Millipore, USA). After blocking, the membranes were incubated with the indicated primary antibody at 4 °C overnight. After washing, the membranes were incubated with horseradish peroxidase-conjugated secondary antibodies for 2 h at 37 °C and visualized by a gel imaging system (Bio-Rad Laboratories, Inc., Hercules, CA, USA).

### Enzyme-linked immunosorbent assay (ELISA)

Following treatment, the macrophages supernatants in each group were collected and centrifuged for 10 min at 1000 rpm. The concentrations of interleukin-1β (IL-1β), interleukin-6 (IL-6), and tumor necrosis factor-α (TNF-α) in diluted serum samples and supernatants of macrophages were examined by immunoassay kit (Jianglai Creatures, China) following the manufacturer’s instructions.

### RNA interference and transfection

Oligonucleotide sequences for RNA interference (RNAi) were as follows: negative control (si-NC), UUCUCCGAACGUGUCACGUTT; FABP4 siRNA, CACUUCCACGAGAGUUUAUTT; Rap1a siRNA, CCAACAGUGUAUGCUCGAATT; JAK2 siRNA, CCACCUGAAUGCAUUGAAATT; SOCS1 siRNA, CCCAGUAUCUUUGCACAAATT. All RNAi oligonucleotides were obtained from Genepharma Company (Shanghai, China). For siRNA transfection, Lipofectamine^®^ 2000 (Invitrogen, Carlsbad, California) was diluted in RPMI 1640 medium and incubated for 5 min at room temperature. Then, siRNA was mixed with Lipofectamine^®^ 2000 and incubated at room temperature for 20 min to form siRNA–Lipofectamine complexes. Finally, the mixture of siRNA and Lipofectamine® 2000 was added into the medium supernatant of macrophages. After incubation for 48 h, the total RNA of the macrophages was extracted and subjected to qRT-PCR analysis.

### Assay of Rap1a activity

Rap1a activity (GTP form) was determined using Rap1 GTP pull-down assay (number ab212011). Briefly, 50% of resin slurry was added to the spin cup with a collection tube. After that, 20 μg of GST-Ral GDS-RBD protein was added to the spin cup containing glutathione resin, followed by 500 μg of cell lysates, and the mixture was incubated in the spin cup at 4 °C. After 1 h of co-incubation, the spin cup was centrifuged and washed three times with the indicated buffer. Finally, to elute the samples, 2× SDS sample buffer was applied to the resin. The eluted samples were subjected to western blot.

### Assay of c-Src protein in the cellular membrane

The macrophages were washed with phosphate-buffered saline three times and broke by ultrasound, and the lysate was centrifuged at 14,000 × *g* and 4 °C for 10 min. Then, the precipitate was mixed with 1 mL of cold extraction buffer, incubated at 37 °C for 10 min, and centrifuged at 14,000 × *g* and 4 °C for 10 min. The obtained samples were divided into upper (cytoplasm protein) and lower layers (membrane proteins). The proteins were subjected to western blot.

### Statistical analysis

All results were analyzed with GraphPad Prism 5.0 software. All error bars in this study represent the mean ± standard deviation (SD) from at least three independent experiments. For western blot and qRT-PCR experiments, we carried out three independent repeated experiments. There were certain differences in the expression and quantitative analysis of these three independent experiments. However, in the statistical analysis, we normalized the data of the treatment group in relation to the control group. Therefore, the statistical graph shows that the value of all samples in the control group is 1. The two groups were compared using the two-tailed Student’s *t* test. Differences between groups were assessed by one-way analysis of variance combined with the Student–Newman–Keuls post-hoc test or the nonparametric Mann–Whitney *U* test. Pearson test was used to analyze the correlation between serum Hcy level and atherosclerotic lesion area. *P* < 0.05 was considered statistically significant.

## Results

### Homocysteine aggravates macrophage inflammation in atherosclerosis of *ApoE*^*−/−*^ mice

**Fig. 1:**
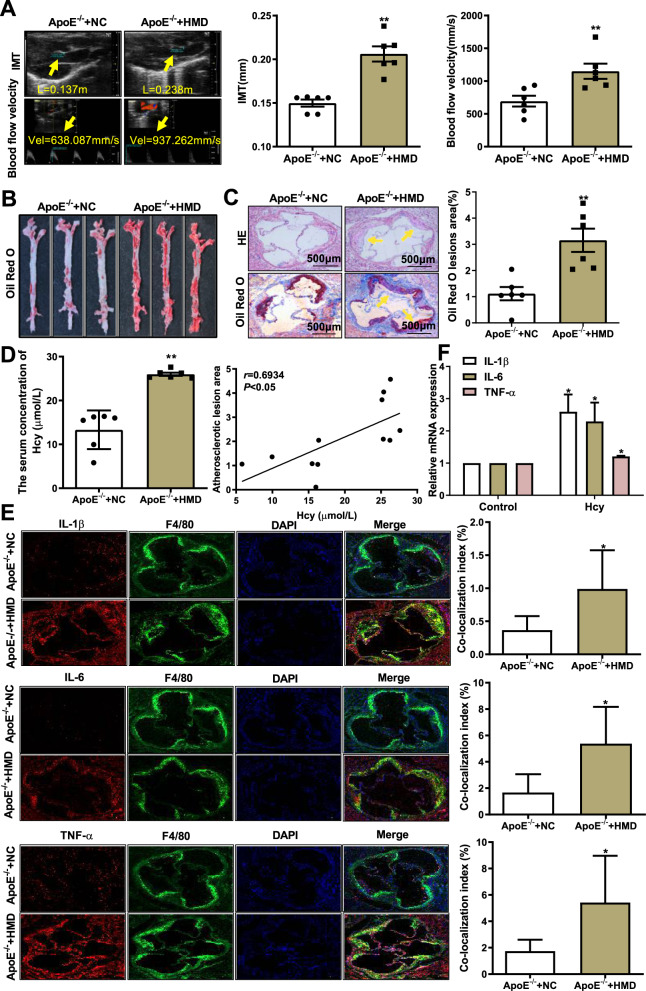
Homocysteine activates macrophage inflammation and facilitates atherosclerosis. **A** Representative images and quantification of ultrasound biomicroscopy of the intima–media thickness (IMT, yellow arrow) of the aortic root and blood velocity (yellow arrow) of the ascending aorta in *ApoE*^*−/−*^ mice fed a high-methionine/normal diet (*n* = 6). **B** Representative images of atherosclerotic plaque at the aortic tree were observed by Oil Red O staining in *ApoE*^*−/−*^ mice (*n* = 6). **C** Representative images of atherosclerotic plaque at the aortic roots were observed by HE and Oil Red O staining. The yellow arrow indicates an atherosclerotic plaque. Scale bar, 200 μm. (*n* = 6). D Levels of serum Hcy in *ApoE*^***−/−***^ mice were measured by an automatic biochemistry analyzer. Correlation between the atherosclerotic plaque area and serum level of Hcy in *ApoE*^*−/−*^ mice fed a high-methionine diet for 16 weeks. Pearson correlation coefficients (*r*) and *P* values are presented in the graphs (*n* = 6). **E** Co-immunofluorescence staining of IL-1β, IL-6, and TNF-α (red) with F4/80 (green, a positive marker of macrophages) in the aortic root of *ApoE*^*−/−*^ mice; nuclei stained with DAPI (blue) (scale bars, 200 μm). **F** The mRNA levels of IL-1β, IL-6, and TNF-α were examined by qRT-PCR after treating macrophages with 100 μmol/L Hcy for 48 h (*n* = 3). Data are expressed as the mean ± SD. *P < 0.05, **P < 0.01, versus *ApoE*^*−/−*^+NC or Control group.

To investigate the possible mechanism of atherosclerosis induced by Hcy, we established an atherosclerosis model as described previously. Ultrasound biomicroscopy results indicated a significant increase in the IMT of the aortic root and ascending aortic blood flow velocity at the arch of the aorta in *ApoE*^*−/−*^ mice fed a high-methionine diet (Fig. 1A). Similarly, HE and Oil Red O staining showed that the plaque area of aortic tree and aortic root were remarkably increased in *ApoE*^*−/−*^ mice fed a high-methionine diet (Fig. 1B and C). Meanwhile, the level of serum Hcy in *ApoE*^*−/−*^ mice fed a high-methionine diet increased, and positively correlated with atherosclerotic lesion area (*r* = 0.6934, *P* < 0.05) (Fig. 1D). These results confirm that Hcy exacerbates atherosclerotic plaque development in *ApoE*^*−/−*^ mice.

IL-1β is mainly secreted by macrophages and can promote the formation of atherosclerosis^17^. IL-6 is an essential pathogenic cytokine for coronary heart disease^18^, and TNF-α is an important proinflammatory cytokine during immune regulation^19^. In addition, a previous study has shown that the pathogenesis of atherosclerosis is related to the expression and secretion of inflammatory molecules, especially IL-1β, IL-6, and TNF-α, by macrophages^20^. To determine whether macrophage inflammation is involved in Hcy-related atherosclerosis, we detected F4/80 (a positive marker of macrophages) and proinflammatory cytokines (IL-1β, IL-6, and TNF-α) in the aortic root of *ApoE*^*−/−*^ mice. The results showed that IL-1β-, IL-6-, and TNF-α-positive macrophages in the aortic root plaque of *ApoE*^*−/−*^ mice fed a high-methionine diet were increased (Fig. 1E). In addition, we found that the mRNA expression levels of these three cytokines were increased in macrophages treated with 100 μmol/L Hcy (Fig. 1F). These results demonstrate that Hcy exacerbates macrophage inflammatory responses in atherosclerosis.

### FABP4 regulates macrophage inflammation induced by Hcy

**Fig. 2:**
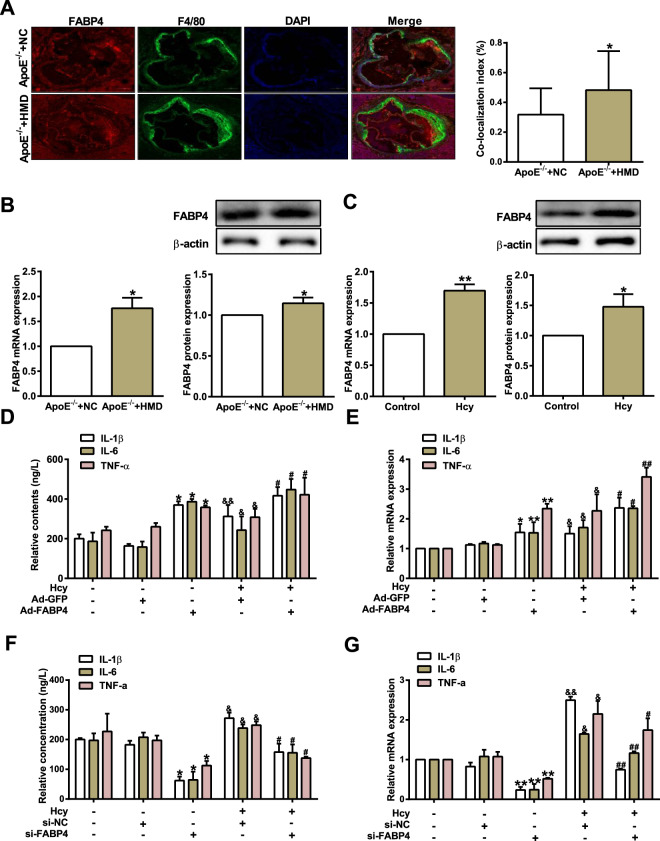
FABP4 facilitates macrophage inflammation induced by homocysteine. **A** Representative immunofluorescence images showing FABP4 (red) co-localization with F4/80 (green) in atherosclerotic aortic root from *ApoE*^*−/−*^ mice. Nuclei were stained with DAPI (blue); scale bars, 200 μm. Quantification of average intensity is shown on the right panel (*n* = 6). **B**, **C** qRT-PCR and western blot detected the mRNA and protein expression levels of FABP4 both in the aortic plaque of *ApoE*^*−/−*^ mice (*n* = 6) and macrophages treated with Hcy (*n* = 3). **D**–**G** The secretion and mRNA levels of IL-1β, IL-6, and TNF-α were measured by ELISA and qRT-PCR in macrophages transfected with Ad-GFP, Ad-FABP4, si-NC, or si-FABP4, followed by treatment with Hcy (*n* = 3). Data are expressed as the mean ± SD. *P < 0.05, **P < 0.01, versus *ApoE*^*−/−*^ + NC, Control group, Ad-GFP or si-NC ^*#*^*P* < 0.05, ^##^*P* < 0.01 versus Ad-GFP + Hcy or si-NC + Hcy group; ^&^*P* < 0.05, ^&&^*P* < 0.01 versus Ad-GFP or si-NC group.

A previous study has demonstrated that FABP4 plays a vital role in Hcy-mediated lipid accumulation^21^. To investigate whether FABP4 participates in macrophage inflammation induced by Hcy, immunofluorescence staining was conducted with antibodies against FABP4 and F4/80 (a positive marker of macrophages). As shown in Fig. 2A, FABP4-positive cells co-localized with F4/80 were notably increased in the aortic root from *ApoE*^*−/−*^ mice fed a high-methionine diet. Likewise, the mRNA and protein expression levels of FABP4 were remarkably increased in the aortic plaque of both *ApoE*^*−/−*^ mice fed a high-methionine diet and macrophages treated with Hcy (Fig. 2B and C). To further confirm the role of FABP4 in the regulation of Hcy-induced macrophage inflammation, we transfected Ad-FABP4 or si-FABP4 into macrophages (Fig. S1A). ELISA and qRT-PCR results showed that the overexpression of FABP4 promoted the secretion and mRNA expression of proinflammatory cytokines IL-1β, IL-6, and TNF-α in macrophages treated with Hcy (Fig. 2D, E). In contrast, knockdown of FABP4 decreased the secretion and mRNA levels of IL-1β, IL-6, and TNF-α (Fig. 2F, G). Taken together, these data suggest that FABP4 promotes macrophage inflammation in response to Hcy.

### JAK2/STAT2 pathway participates in FABP4-activated macrophage inflammation induced by Hcy

**Fig. 3:**
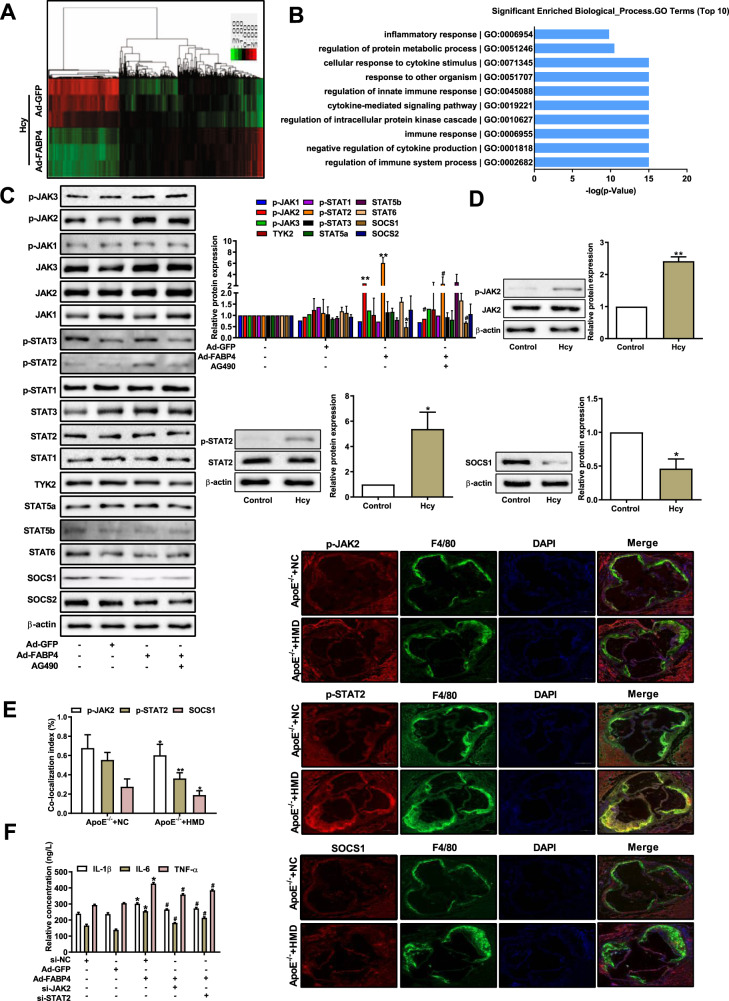
FABP4 promotes macrophage inflammation due to homocysteine treatment via the JAK2/STAT2 pathway. **A** The heat map of 1751 differentially expressed genes (DEGs, microarray fold change ≤0.5 or ≥2, unpaired *t*-test *P* ≤ 0.05, *n* = 3) in macrophages transfected with Ad-FABP4 and treated with Hcy. Red represents upregulated genes and green represents downregulated genes. **B** The top ten enriched Gene Ontology (GO) analysis of the DEGs and functional comment in macrophages transfected with Ad-FABP4 and treated with Hcy. **C** Western blot analysis of JAKs, STATs, and SOCSs in FABP4-overexpressing macrophages with or without JAK2 inhibitor (AG490), treated with Hcy (*n* = 3). **D** Western blot analysis of p-JAK2, p-STAT2, and SOCS1 in macrophages treated with Hcy (*n* = 3). **E** Representative immunofluorescence images and the intensity of p-JAK2, p-STAT2, and SOCS1 (red) co-localized with F4/80 (green) in *ApoE*^*−/−*^ mice. Nuclei were stained with DAPI (blue) (scale bars, 200 μm) (*n* = 6). **F** ELISA was used to analyze the concentrations of IL-1β, IL-6, and TNF-α in FAPB4-overexpressing macrophages transfected with si-JAK2 or si-STAT2 and treated with Hcy (*n* = 3). Data are expressed as the mean ± SD. *P < 0.05, **P < 0.01, versus *ApoE*^*−/−*^ + NC, Control, or Ad-GFP + Hcy group; ^#^*P* < 0.05 versus Ad-FABP4 + Hcy or si-NC + Ad-FABP4 + Hcy group.

To further determine which pathway is involved in FABP4-mediated macrophage inflammation, gene chip was used to identify differentially expressed genes (DEGs) in FABP4-overexpressing macrophages treated with Hcy. The results showed that 1751 genes were differentially expressed (microarray fold change ≥2 and *P* < 0.05) (Fig. 3A). Then, the regulatory DEGs were extracted for Gene Ontology (GO) enrichment analysis by R language. According to GO pathway analysis, these DEGs were strongly related to cellular response to cytokine stimulus, regulation of immune system process, inflammatory response, and regulation of glucose and lipid homeostasis based on microarray data. Moreover, we found that these DEGs were enriched in inflammation-related pathways (Fig. 3B). Specifically, 21 DEGs were mainly associated with JAK/STAT/SOCS pathway, including significant upregulation of JAK2 and STAT1/2 and downregulation of SOCS1 (Table 2). Accordingly, western blot results showed that the phosphorylation of JAK2 and STAT2 (p-JAK2, p-STAT2) significantly increased, while SOCS1 decreased in FABP4-overexpressing macrophages treated with Hcy (Fig. 3C).

**Table 2:** Microarray analysis of the downstream genes of macrophages overexpressing FABP4.

Rank	Gene ID	Score(d)	*q*-value (%)	Fold change	Public ID
1	11762533_a_at	29.26805224	0	664.5195	Rap1a
2	11728039_s_at	28.22943064	0	228.4381	CCL8
3	11720298_at	25.95233949	0	115.2291	CXCL10
4	11717686_a_at	−3.202034435	0.059747478	0.6634	JAK1
5	11756857_a_at	4.462438982	0	4.63	JAK2
6	11761631_at	−0.735334842	15.423159	0.8765	JAK3
7	11726689_a_at	11.70086601	0	6.8003	STAT1
8	11755147_s_at	10.69343787	0	3.9107	STAT2
9	11720756_at	1.326537621	2.587459719	1.3577	STAT3
10	11745364_a_at	−0.26769549	31.28237274	0.9571	STAT5a
11	11717178_a_at	0.101723713	34.21585509	1.0182	STAT5a
12	11720229_a_at	−1.05340244	7.712832008	0.8447	STAT5b
13	11720228_at	−1.233059781	5.359290918	0.7812	STAT5b
14	11742723_a_at	1.104719786	4.592240312	1.2886	STAT6
15	11742724_x_at	1.602943185	1.110300781	1.3297	STAT6
16	11746850_a_at	0.896573247	8.125504819	1.1754	STAT6
17	11753889_a_at	−0.017378262	37.31611208	0.9986	STAT6
18	11737146_a_at	8.07839487	0	2.4483	SOCS1
19	11719218_at	−0.160467809	34.83399354	0.9763	SOCS3
20	11729319_a_at	1.844556344	0.605324626	1.6249	SOCS5
21	11723362_s_at	2.266218531	0.203831936	2.4913	SOCS6

AG490, an inhibitor of JAK2, was used to confirm the effect of JAK2 and STAT2 in FABP4-overexpressing macrophages treated with Hcy. AG490 obviously reduced the levels of p-JAK2 and p-STAT2 and increased the level of SOCS1, suggesting that the JAK2/STAT2/SOCS1 pathway was activated in FABP4-overexpressing macrophages. In addition, we observed that Hcy significantly increased the levels of p-JAK2 and p-STAT2, while it decreased SOCS1 expression in macrophages treated with Hcy (Fig. 3D). Consistent with western blot, co-immunofluorescence staining showed that more p-JAK2- and p-STAT2-positive cells co-localized with F4/80 in the aortic plaque from *ApoE*^*−/−*^ mice fed a high-methionine diet than in that from *ApoE*^−/−^ mice fed a regular diet, while SOCS1 decreased (Fig. 3E). Furthermore, we found that knockdown of JAK2 or STAT2 significantly alleviated the promotion effect of FABP4 on Hcy-treated macrophages (Figs. 3F and  S1B). In conclusion, these data suggest that FABP4 promotes macrophage inflammation induced by Hcy via the activation of the JAK2/STAT2/SOCS1 pathway.

### Rap1a is required for FABP4-dependent activation of the JAK2/STAT2 pathway in macrophage inflammation induced by Hcy

**Fig. 4:**
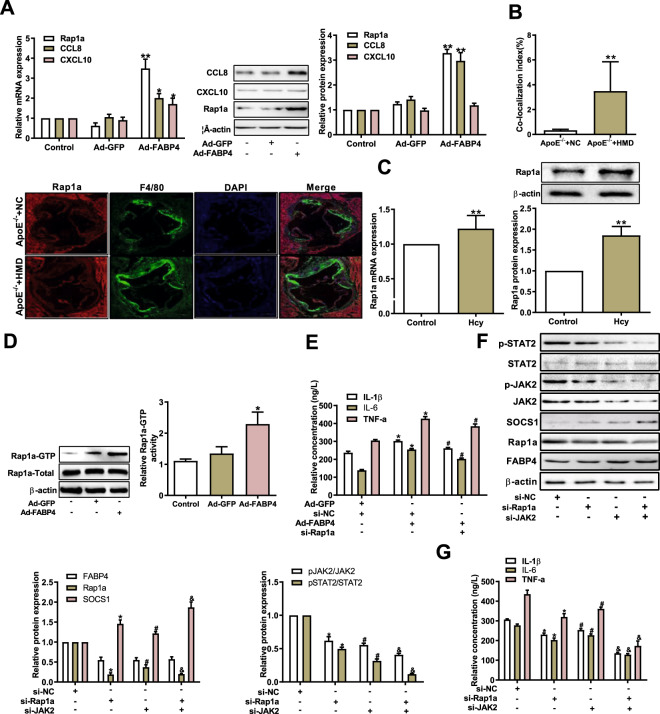
FABP4 regulates the JAK2/STAT2 pathway via Rap1a in macrophage inflammation. **A** The mRNA and protein expression levels of Rap1a, CCL8, and CXCL10 in macrophages were analyzed by qRT-PCR and western blot after transfection with Ad-GFP and Ad-FABP4 and treatment with Hcy (*n* = 3). **B** Representative immunofluorescence images and qualification of the co-localization of Rap1a (red) and F4/80 (green) in the aortic root from *ApoE*^*−/−*^ mice. Nuclei were stained with DAPI (blue) (scale bars, 50 μm) (*n* = 6). **C** The mRNA and protein expression levels of Rap1a were examined in macrophages treated with Hcy (*n* = 3). **D** Rap1a-GTP (activated Rap1a) level was measured in macrophages transfected with Ad-FABP4 and treated with Hcy. The total Rap1a protein level was used as loading control (*n* = 3). **E** The concentrations of IL-1β, IL-6, and TNF-α were analyzed by ELISA in macrophages transfected with Ad-FABP4 and/or si-Rap1a treated with Hcy (*n* = 3). **F** The protein expression levels of FABP4, Rap1a, p-JAK2, p-STAT2, and SOCS1 were analyzed by western blot in FABP4-overexpressing macrophages transfected with si-Rap1a and/or si-JAK2 and treated with Hcy (n = 3). **G** The concentrations of IL-1β, IL-6, and TNF-α were examined by ELISA in macrophages described as above (*n* = 3). Data are expressed as the mean ± SD. *P < 0.05, **P < 0.01, versus *ApoE*^*−/−*^ + NC, Ad-GFP + Hcy, Ad-FABP4 + Hcy, or Ad-FABP4 + si-NC + Hcy group; ^#^*P* < 0.05 versus si-NC + Ad-FABP4 + Hcy group; ^&^*P* < 0.05 versus Ad-FABP4 + si-JAK2 + Hcy group.

Further analysis of the gene chip indicated that CCL8, CXCL10, and Rap1a—the top three of all the DEGs (Table 2)—were dramatically upregulated in FABP4-overexpressing macrophages treated with Hcy. Consistent with the gene chip analysis, a significant upregulation of Rap1a and CCL8 expression was observed in FABP4-overexpressing macrophages treated with Hcy (Fig. 4A). To determine whether Rap1a participates in macrophage inflammation in atherosclerosis, immunofluorescence staining showed that more Rap1a-positive cells co-localized with F4/80 in the aortic plaque of *ApoE*^*−/−*^ mice fed a high-methionine diet than in that from *ApoE*^−/−^ mice fed a regular diet (Fig. 4B). Likewise, the mRNA and protein expression levels of Rap1a were obviously increased in macrophages treated with Hcy (Fig. 4C). As a member of the small GTPase in the Ras family, Rap1a usually circulates between the inactive GDP-bound state and the active GTP-bound state to play a role in regulating cell functions. As expected, Rap1a was activated in FABP4-overexpressing macrophages (Fig. 4D). Meanwhile, to further investigate the relationship between FABP4 and Rap1a, Ad-FABP4 and si-Rap1a were co-transfected into macrophages. We found that FABP4 overexpression apparently enhanced the levels of proinflammatory cytokines, which was reversed by knockdown of Rap1a in macrophages treated with Hcy (Figs. S2 and  4E), implying that Rap1a is a critical mediator in FABP4-induced macrophage inflammation after treatment with Hcy.

To further clarify the function of Rap1a in FABP4-dependent activation of the JAK2/STAT2/SOCS1 pathway in Hcy-induced macrophage inflammation, Ad-FABP4 was co-transfected with si-Rap1a or si-JAK2 into macrophages. The results showed that knockdown of Rap1a or JAK2 decreased the expression of p-JAK2 and p-STAT2 in FABP4-overexpressing macrophages. Simultaneously, the combination of knockdown of Rap1a and JAK2 further restrained p-JAK2 and p-STAT2 expression, and increased the SOCS1 expression (Fig. 4F), compared with simple knockdown Rap1a or JAK2. The combination of knockdown of Rap1a and JAK2 reduced the secretion of proinflammatory factors IL-1β, IL-6, and TNF-α in FABP4-overexpressing macrophages treated with Hcy compared with simple knockdown of Rap1a or JAK2 (Fig. 4G). These results demonstrate that Rap1a is a mediator of the FABP4-regulated JAK2/STAT2/SOCS1 pathway in macrophage inflammation induced by Hcy.

### The phosphorylation of c-Src at Tyr416 is a key to FABP4-dependent activation of the JAK2/STAT2 pathway in macrophage inflammation induced by Hcy

**Fig. 5:**
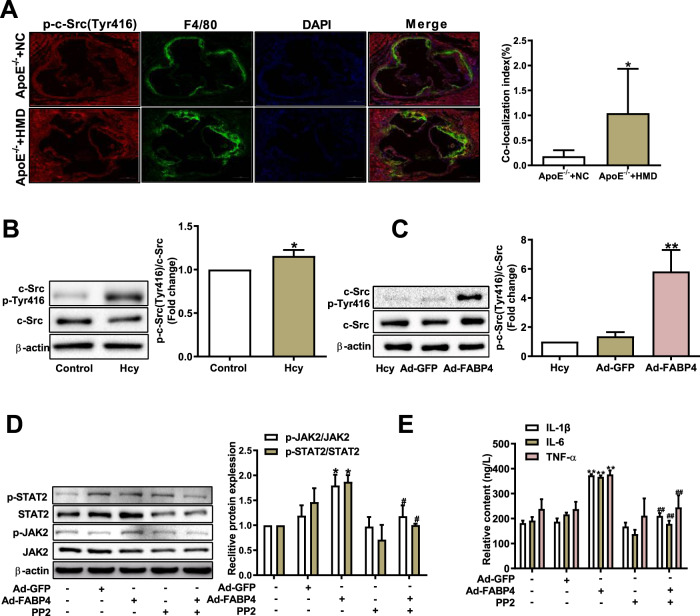
FABP4 activates the JAK2/STAT2 pathway through c-Src in macrophages treated with homocysteine. **A** The co-localization of p-Tyr416 c-Src (red) and F4/80 (green) in the aortic root of *ApoE*^*−/−*^ mice; nuclei stained with DAPI (blue) (scale bars, 200 μm) (*n* = 6). **B** Western blot analysis of the p-Tyr416 c-Src in macrophages treated with Hcy (*n* = 3). **C** The p-Tyr416 c-Src was determined in macrophages transfected with Ad-FABP4 and treated with Hcy (*n* = 3). **D** Western blot analysis of the expression of p-JAK2 and p-STAT2 in FABP4-overexpressing macrophages after pretreatment with c-Src inhibitor PP2 and treatment with Hcy (*n* = 3). **E** The concentrations of IL-1β, IL-6, and TNF-α were examined by ELISA in macrophages described above (*n* = 3). Data are expressed as the mean ± SD. *P < 0.05 versus *ApoE*^*−/−*^ + NC or Control group, *P < 0.05, **P < 0.01 versus Ad-GFP + Hcy group; ^#^*P* < 0.05, ^##^*P* < 0.01 versus Ad-FABP4^+^Hcy group.

c-Src, a non-receptor tyrosine kinase, plays a critical role in transducing intracellular signals from the receptors^22^. To illustrate whether c-Src is involved in the regulation of Hcy-induced macrophage inflammation, immunofluorescence staining was used to measure Tyr416 phosphorylation of c-Src (p-Tyr416 c-Src, the activated form of c-Src) in *ApoE*^*−/−*^ mice. We found an increased proportion of p-Tyr416 c-Src-positive cells in macrophages of *ApoE*^*−/−*^ mice fed a high-methionine diet (Fig. 5A). Similarly, the expression of p-Tyr416 c-Src was also dramatically increased in macrophages treated with Hcy (Fig. 5B). Next, we sought to determine the role of c-Src in the activation of JAK2/STAT2 by FABP4 in Hcy-induced macrophage inflammation. Notably, the overexpression of FABP4 increased the Tyr416 phosphorylation of c-Src (Fig. 5C). Furthermore, PP2—a c-Src family kinase inhibitor—obviously inhibited the phosphorylation of JAK2 and STAT2 in response to FABP4 (Fig. 5D). Meanwhile, the overexpression of FABP4 apparently enhanced the secretion of proinflammatory factors IL-1β, IL-6, and TNF-α, while PP2 reversed the effect (Fig. 5E). These results indicate that the Tyr416 phosphorylation of c-Src in macrophages treated with Hcy is critically beneficial for FABP4-induced JAK2/STAT2 pathway activation.

### FABP4 promotes membrane translocation of c-Src and its p-Tyr416 via Rap1a

**Fig. 6:**
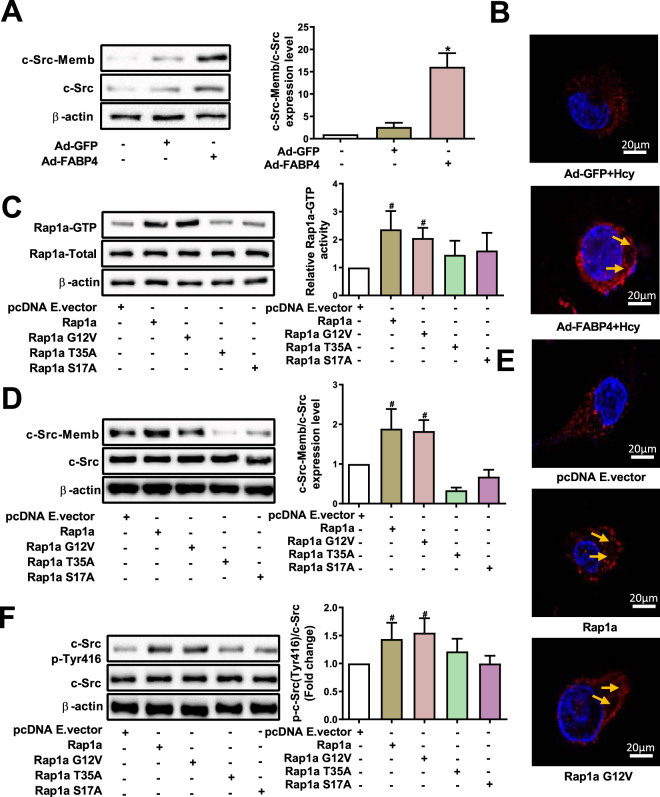
Rap1a induces c-Src membrane translocation and its phosphorylation at Tyr416 in macrophages. **A** Total and membrane protein levels of c-Src in FABP4-overexpressing macrophages treated with Hcy were analyzed by western blot (*n* = 3). **B** The localization of p-Tyr416 c-Src (red) was detected by immunofluorescence. Nuclei were stained with DAPI (blue). The yellow arrow indicates macrophage membrane (scale bars, 20 μm) (*n* = 3). **C** The levels of GTP-bound form of Rap1a were detected in macrophages after transfection with several putative Rap1a dominant-positive or -negative mutants (Rap1a-G12V, Rap1a-T35A, and Rap1a-S17A mutants) (*n* = 3). **D** The total and membrane protein levels of c-Src were tested in macrophages after co-transfecting with several putative Rap1a dominant-negative or -positive mutants (Rap1a-G12V, Rap1a-T35A, and Rap1a-S17A mutants) upon Ad-FABP4 transduction and Hcy stimulation (*n* = 3). **E** The localization of p-Tyr416 c-Src (red) in macrophages transfected with Rap1a-, Rap1a-G12V-expressing vectors, or the pcDNA empty vector (pcDNA E. vector) was detected by immunofluorescence. Nuclei were stained with DAPI (blue). The yellow arrow indicates the macrophage membrane (scale bars, 20 μm) (*n* = 3). **F** The p-Tyr416 levels of c-Src were tested by western blot in macrophages after transfection with several putative Rap1a dominant-positive or -negative mutants (Rap1a-G12V, Rap1a-T35A, and Rap1a-S17A mutants) upon Ad-FABP4 transduction and Hcy stimulation (*n* = 3). Data are expressed as the mean ± SD. *P < 0.05 versus Ad-GFP + Hcy group; ^#^*P* < 0.05 versus pcDNA E. vector+Ad-FABP4 + Hcy group.

To explore whether FABP4 affects membrane translocation of c-Src, we detected protein levels of total c-Src and membrane c-Src after FABP4 overexpression in macrophages. As shown in Fig.6A, FABP4 noticeably increased the level of membrane c-Src. Moreover, immunofluorescence results indicated that the p-Tyr416 level of c-Src and its membrane localization were markedly enhanced by FABP4 (Fig. 6B). We next sought to investigate the impact of Rap1a activation on the FABP4-induced membrane translocation of c-Src. First, several putative dominant-negative and positive mutants of Rap1a were constructed and transfected into macrophages. As shown in Fig. 6C, macrophages transfected with Rap1a- or its dominant-positive mutant-expressing vectors (Rap1a-G12V) had higher levels of the activated form of Rap1a (Rap1a-GTP), whereas no significant alteration in Rap1a-GTP levels was observed in macrophages transfected with dominant-negative mutants of Rap1a (Rap1a-T35A, Rap1a-S17A). Subsequently, Rap1a and its mutants were used to assess whether FABP4 activated c-Src via Rap1a in macrophages treated with Hcy, which in turn modulated the membrane translocation of c-Src. Strikingly, we found that the membrane translocation of c-Src was significantly increased by Rap1a- or the dominant-positive mutant (Rap1a-G12V), but not the dominant-negative mutants (Rap1a-T35A, Rap1a-S17A), in FABP4-overexpressing macrophages treated with Hcy (Fig. 6D, E). Correspondingly, the level of p-Tyr416 c-Src also remarkably increased in macrophages transfected with Rap1a- or Rap1a-G12V mutant-expressing vectors (Fig. 6F). These results suggest a central role of Rap1a in FABP4-dependent activation of c-Src in macrophages treated with Hcy.

### SOCS1 has a negative regulatory role via Rap1a in FABP4-dependent activation of the JAK2/STAT2 pathway induced by Hcy

**Fig. 7:**
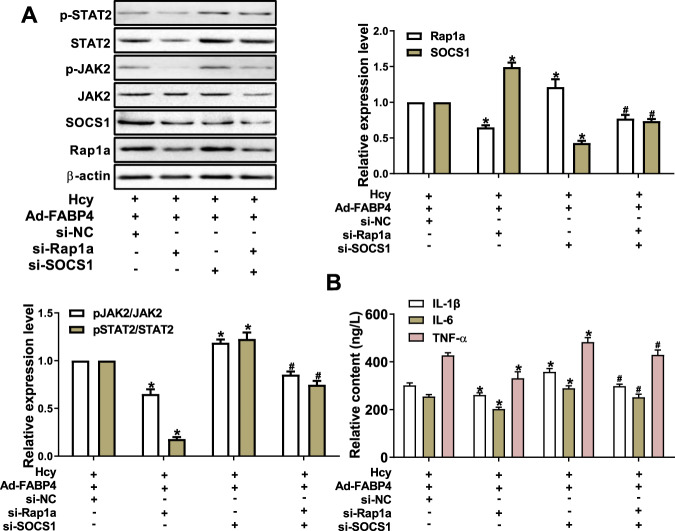
SOCS1 inhibits the expression of Rap1a and activation of the JAK2/STAT2 pathway in macrophage inflammation induced by homocysteine. **A** Western blot analysis of Rap1a, p-JAK2, p-STAT2, and SOCS1 in Ad-FABP4 macrophages transfected with si-Rap1a and/or si-SOCS1 and treated with Hcy (*n* = 3). **B** The concentrations of IL-1β, IL-6, and TNF-α were analyzed by ELISA in macrophages as described above (*n* = 3). Data are expressed as the mean ± SD. *P < 0.05 versus Ad-FABP4 + si-NC + Hcy group; ^#^*P* < 0.05 versus Ad-FABP4 + si-Rap1a+Hcy group.

To further elucidate the regulatory role of SOCS1 in the JAK2/STAT2 pathway and Rap1a expression, we knocked down SOCS1 in FABP4-overexpressing macrophages (Fig. S3). The results showed that the expression levels of p-JAK2, p-STAT2, and Rap1a increased, which was reversed by knockdown of Rap1a (Fig. 7A), Based on the aforementioned results, SOCS1 could suppress the expression of Rap1a and activation of the JAK2/STAT2 pathway in FABP4-overexpressing macrophages treated with Hcy. In addition, knockdown of SOCS1 increased the secretion of proinflammatory factors by FABP4-overexpressing macrophages, which was reversed by knockdown of Rap1a (Fig. 7B). Taken together, SOCS1 has a negative regulatory role in FABP4-dependent activation of the JAK2/STAT2 pathway and Rap1a in macrophage inflammation induced by Hcy.

### Discussion

As a chronic inflammatory disorder of the arterial vessel wall, atherosclerosis is one of the leading causes of death worldwide^23^. Multiple risk factors have been identified, including hyperlipoproteinemia, hypertension, diabetes, obesity, and smoking^24^. At present, Hcy is considered an independent risk factor of atherosclerosis^25^. It has been reported that Hcy accelerates atherogenic process and causes vascular inflammation by inducing the expression of inflammatory genes^26^. In this study, we found that Hcy aggravated macrophage inflammation in atherosclerosis. Although it has been shown that Hcy has proinflammatory effects in atherosclerosis, the exact cellular and molecular mechanisms remain obscure.

FABP4 has been linked to various physiological and pathological processes, such as inflammatory regulation and lipid metabolism^27^. It is traditionally considered a cytoplasmic protein that transports free fatty acids to specific enzymes and cell compartments, thereby playing an important role in inflammatory response^28^. A previous study has found that peroxisome proliferator-activated receptor γ activity is enhanced and proinflammatory pathways are suppressed in the absence of FABP4 in macrophages^29^. Other studies have shown that the loss of FABP4 can inhibit the inflammatory signaling in macrophages, which is a major reason for the metabolic benefits of the lack of FABP4^30^. Notably, knockdown of FABP4 in macrophages significantly prevents the development of atherosclerosis^31^. It has been previously reported that FABP4 regulates macrophage inflammation via oxidative stress^32^. In addition, Hcy leads to oxidative stress, which interferes with different cellular signaling pathways in atherosclerosis^30^, suggesting that FABP4 may have a similar effect in macrophage inflammation induced by Hcy.

The JAK/STAT pathway is a widely expressed intracellular signal transduction pathway; it correlates with macrophage inflammation in atherosclerosis^33^. JAK1, JAK2, JAK3, and TYK2 are members of the JAK family, while STAT1, STAT2, STAT3, STAT5A/B, and STAT6 are members of the STAT family^34^. It has been reported that the JAK/STAT pathway switches on and releases inflammatory cytokines, such as IL-1β, TNF-α, and IL-6 in LPS-stimulated RAW 264.7 cells^35^. Meanwhile, FABP4 directly impairs the functions of human coronary endothelial cells through the upregulation of the ERK/JNK/STAT1 pathways and the downregulation of the eNOS and SDF-1 pathways^36^. In this study, we found that FABP4 facilitated macrophage inflammation induced by Hcy through activating the JAK2/STAT2 pathway. In addition, the SOCS family, a negative regulator of the JAK/STAT pathway, comprises cytokine-inducible SH2 domain-containing protein and SOCS1–7, which are involved in cell proliferation, differentiation, apoptosis, and immune regulation^8,37^. A previous study has found that SOCS1 and SOCS3 lead to sustained cytokine activation of STATs and contribute to the progression of inflammation^38^. Similar results were obtained in our research, where SOCS1 showed a negative regulatory role in FABP4-dependent activation of the JAK2/STAT2 pathway in Hcy-induced macrophage inflammation. According to our results, not only can FABP4 cause atherogenesis through the activation of inflammatory factors, including IL-1β, IL-6, and TNF-α, but it can also lead to macrophage inflammation through the activation of the JAK2/STAT2 pathway, which in turn may be regulated by SOCS1.

Rap1a is a member of the small GTPases of the Ras family, and is involved in effector-induced phagocytosis regulation in macrophages^39^. There is growing evidence that Rap1 produced in excess as a cause for vascular remodeling and inflammation leads to the progression of atherosclerosis^40^. According to our gene chip analysis, Rap1a is involved in the FABP4-mediated Hcy-induced macrophage inflammation. In addition, Hcy has been demonstrated to play a role in oxidative stress, which can affect the Rap1 levels, suggesting that Rap1a is increased in FABP4-mediated macrophage inflammation induced by Hcy^41^. The underlying mechanisms related to inflammation in the Ras family involve increased proinflammatory TNF-α and NF-κB pathway activation or elevated inflammatory receptor expression^42^. Circulating levels of large extracellular vesicles carrying Rap1 induce vascular smooth muscle cells migration and proliferation by a mechanism involving the Rap1/ERK5 axis and proinflammatory profile^43^. Surprisingly, Rap1a regulated the JAK2/STAT2 pathway in Hcy-induced macrophage inflammation. Ras and its related small GTPases are important signaling nodes that regulate a wide variety of cellular functions. The active form of these proteins exists in a transient GTP-bound state that mediates downstream signaling events^44^. Moreover, we also found that FABP4 overexpression promoted the active GTP-binding form of Rap1a in macrophages treated with Hcy. Our study confirmed that Rap1a was necessary for FABP4-dependent activation of the JAK/STAT pathway in Hcy-induced macrophage inflammation.

In addition, tyrosine kinase c-Src has been implicated in a variety of cellular responses, including inflammation^45^. Hcy directly or indirectly participates in cell signal transduction by oxidative stress, which is induced by increasing the intracellular concentration of reactive oxygen species^46^. Moreover, c-Src phosphorylation at multiple sites, including Tyr416 and Tyr527, can directly regulate downstream target genes^47^. In this study, we found that the level of p-Tyr416 c-Src significantly increased in FABP4-overexpressing macrophages treated with Hcy, confirming the critical role of c-Src phosphorylation in FABP4-mediated macrophage inflammatory responses induced by Hcy. The JAK/STAT pathways are classical signaling pathways in inflammatory response; they become activated by tyrosine phosphorylation in response to various effectors^48^. It has been reported that c-Src can regulate the activation of JAK/STAT in endothelial cells, and the suppression of c-Src and JAK activity can result in STAT dysfunction in human breast cancer cells^49,50^. Similarly, we found that the inhibition of c-Src activation almost eliminated Rap1a-induced elevation of p-JAK2 and p-STAT2 in macrophages treated with Hcy, thereby inhibiting macrophage inflammatory response induced by Hcy. These results suggest that c-Src is required for Rap1a-induced JAK2/STAT2 pathway activation in Hcy-induced macrophage inflammation. More importantly, c-Src, a myristoylated protein, localizes both within and outside lipid rafts before translocation to the plasma membrane^51^. Evidence from other studies suggests that c-Src activated through the plasma membrane transposition can transmit cellular signaling^52^. This study also showed that Rap1a promoted the Hcy-induced c-Src translocation in macrophages, further explaining the mechanism of the FABP4-dependent activation of the JAK2/STAT2 pathway through Rap1a in Hcy-induced macrophage inflammation.

Collectively, our results demonstrated that FABP4 activated the JAK2/STAT2 pathway in macrophage inflammatory response induced by Hcy. We further found that FABP4 promoted c-Src phosphorylation at Tyr416 and membrane translocation via Rap1a, which contributed to the accelerated macrophage inflammation induced by Hcy. Thus, these findings provide a perspective on therapeutic opportunities and challenges for FABP4 in atherosclerosis.

## Supplementary information

Supplementary material

## Data Availability

The datasets used and analyzed in this study are available from the corresponding author on reasonable request.
