# Supplementary material for: FABP4 activates the JAK2/STAT2 pathway via Rap1a in the homocysteine-induced macrophage inflammatory response in ApoE−/− mice atherosclerosis
Source: Lab Invest. 2021 Nov 1;102(1):25–37. doi: 10.1038/s41374-021-00679-2 (PMC8695379; doi:10.1038/s41374-021-00679-2)
Supplement: Supplementary file 1 — Supplementary material [file 41374_2021_679_MOESM1_ESM.pdf]

Supplementary Figures

Figure S1. The mRNA and protein expression of FABP4, JAK2 and STAT2

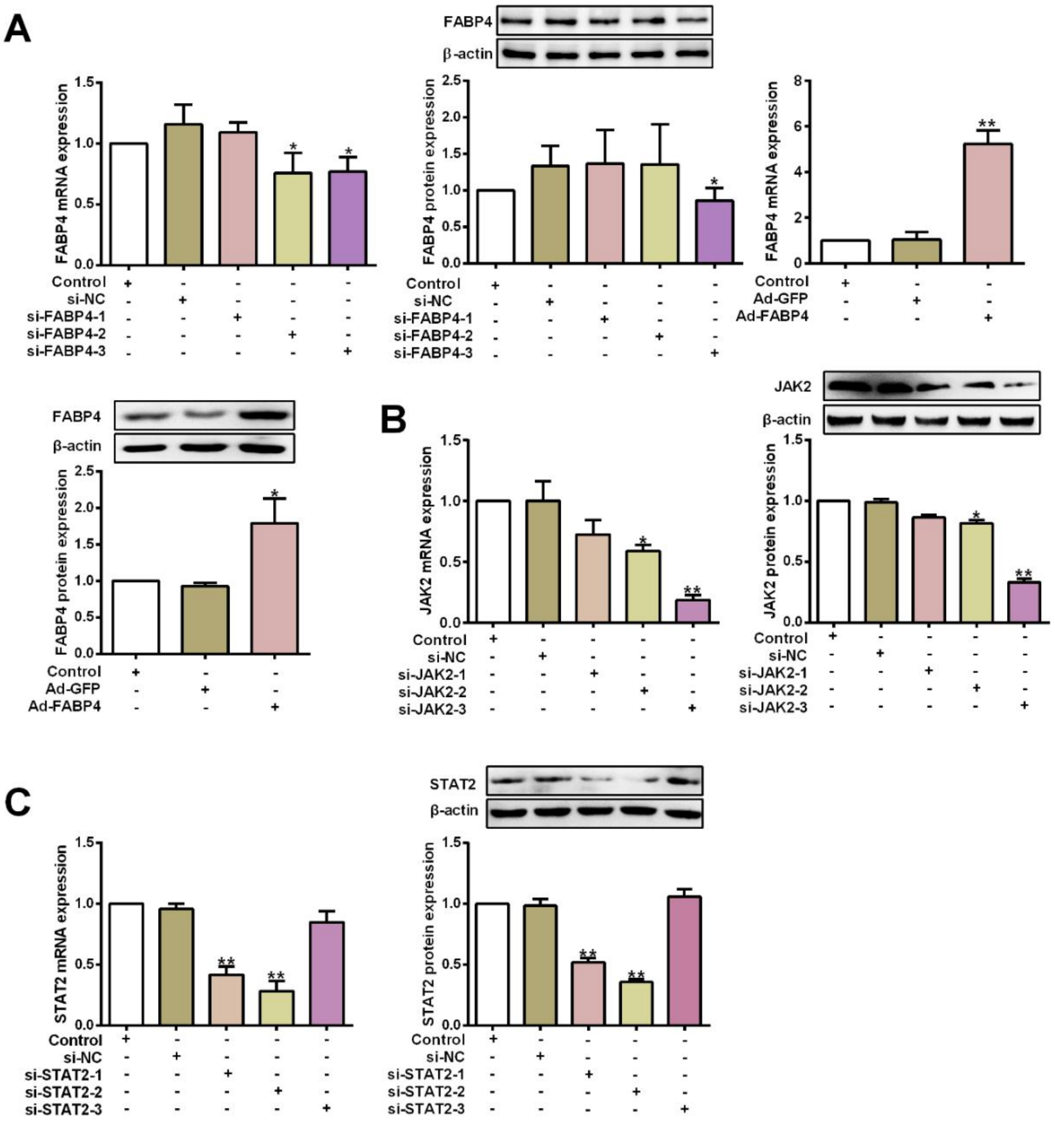

Supplementary Figure 1. The mRNA and protein expression of FABP4, JAK2 and STAT2. (A) The mRNA and protein level of FABP4 were examined by qRT-PCR and western blot in macrophages after infection with adenovirus over-expressing FABP4 (Ad-FABP4) or si-FABP4. (B) JAK2 were silenced by three different siRNAs and their mRNA and protein expression were measured by real-time PCR and western blot in macrophages. (C) STAT2 were silenced by three different siRNAs and their mRNA and protein expression were measured by real-time PCR and western blot in macrophages. Data represent the Mean ± SD of three separate experiments. $^{*}P<$

0.05, $^{**}P< 0.01$ versus Ad-GFP group or si-NC group.

Figure S2. The mRNA and protein expression of Rap1a

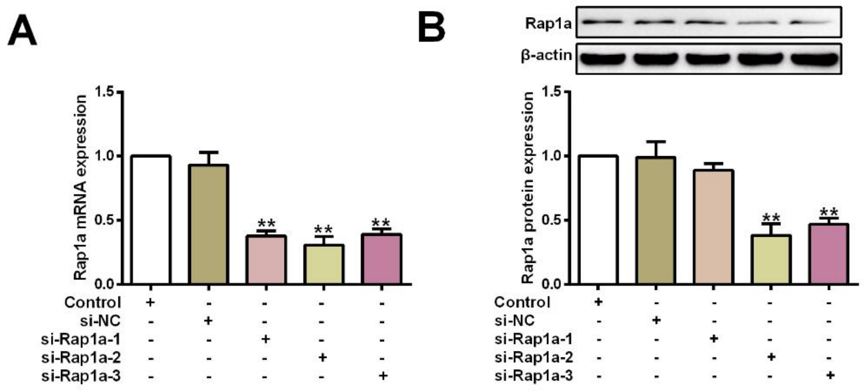

Supplementary Figure 2. The mRNA and protein expression of Rap1a. (A, B) The Rap1a expression was measured by qRT-PCR and western blot after cells were transfected with three different siRNAs (si-Rap1a-1, 2 and 3) of Rap1a in macrophages, respectively. Data represent the Mean±SD of three separate experiments. $^{**}P< 0.01$ versus si-NC group.

Figure S3. The mRNA and protein expression of SOCS1

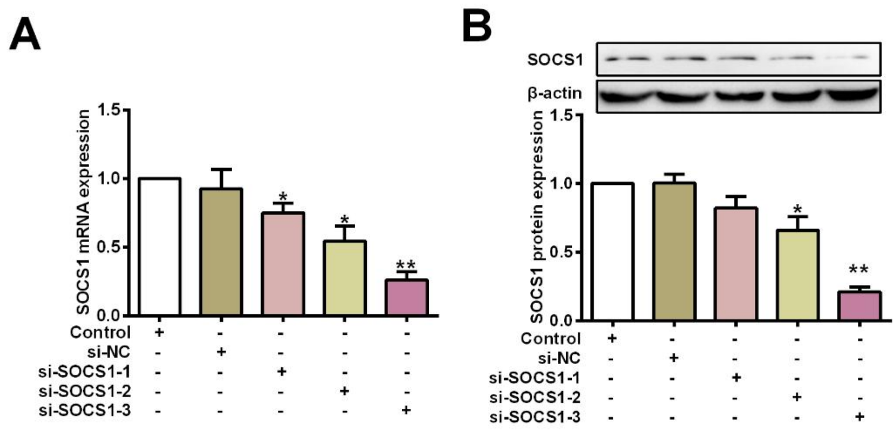

Supplementary Figure 3. The mRNA and protein expression of SOCS1. (A, B) The SOCS1 expression was measured by qRT-PCR and western blot after cells were transfected with three different siRNAs (si-SOCS1-1, 2 and 3) of SOCS1 in macrophages, respectively. Data represent the Mean±SD of three separate experiments. $^{*}P< 0.05$, $^{**}P< 0.01$ versus si-NC group.
